# Mitophagy and cancer: role of BNIP3/BNIP3L as energetic drivers of stemness features, ATP production, proliferation, and cell migration

**DOI:** 10.18632/aging.205939

**Published:** 2024-06-04

**Authors:** Marta Mauro-Lizcano, Federica Sotgia, Michael P. Lisanti

**Affiliations:** 1 Translational Medicine, School of Science, Engineering and Environment (SEE), University of Salford, Greater Manchester, United Kingdom

**Keywords:** mitophagy, BNIP3, BNIP3L(NIX), cancer stem cells (CSCs), flow cytometry (FACS)

## Abstract

Mitophagy is a selective form of autophagy which permits the removal of dysfunctional or excess mitochondria. This occurs as an adaptative response to physiological stressors, such as hypoxia, nutrient deprivation, or DNA damage. Mitophagy is promoted by specific mitochondrial outer membrane receptors, among which are BNIP3 and BNIP3L. The role of mitophagy in cancer is being widely studied, and more specifically in the maintenance of cancer stem cell (CSC) properties, such as self-renewal. Given that CSCs are responsible for treatment failure and metastatic capacity, targeting mitophagy could be an interesting approach for CSC elimination. Herein, we describe a new model system to enrich sub-populations of cancer cells with high basal levels of mitophagy, based on the functional transcriptional activity of BNIP3 and BNIP3L. Briefly, we employed a BNIP3(L)-promoter-eGFP-reporter system to isolate cancer cells with high BNIP3/BNIP3L transcriptional activity by flow cytometry (FACS). The model was validated by using complementary lysosomal and mitophagy-specific probes, as well as the mitochondrially-targeted red fluorescent protein (RFP), namely mt-Keima. High BNIP3/BNIP3L transcriptional activity was accompanied by increases in i) BNIP3/BNIP3L protein levels, ii) lysosomal mass, and iii) basal mitophagy activity. Furthermore, cancer cells with increased BNIP3/BNIP3L transcriptional activity exhibited CSC features, such as greater mammosphere-forming ability and high CD44 levels. To further explore the model, we also analysed other stemness characteristics in MCF7 and MDA-MB-231 breast cancer cell lines, directly demonstrating that BNIP3(L)-high cells were more metabolically active, proliferative, migratory, and drug-resistant, with elevated anti-oxidant capacity. Therefore, high levels of basal mitophagy appear to enhance CSC features.

## INTRODUCTION

Mitophagy is a selective form of macro-autophagy in which unwanted or damaged mitochondria are preferentially targeted for degradation at the auto-phago-lysosome, as an adaptative response to physiological stressors, such as hypoxia, nutrient deprivation, and/or DNA damage. As such, mitophagy represents an important mitochondrial quality control mechanism, as it preserves mitochondrial function, limits the production of damaging reactive oxygen species (ROS) and ensures the efficient use of scarce metabolites and oxygen [1]. Mitophagy plays an important role in many cellular processes, such as embryonic development, cell differentiation, inflammation, and apoptosis. Consequently, defects in mitophagy have been linked to various pathological conditions, including neuro-degeneration, heart failure, cancer, and aging [2].

Mitophagy is promoted by specific mitochondrial outer membrane receptors, or ubiquitin molecules conjugated to proteins on the mitochondrial surface, which interact directly with processed LC3/GABARAP proteins, leading to the formation of auto-phagosomes surrounding the mitochondria. These mitophagy receptors and modulators include: Parkin, FUNDC1, BNIP3, BNIP3L (NIX), and/or p62/SQSTM1 [3].

One of the major molecular mechanisms that regulates mitophagy is the BNIP3/BNIP3L-dependent pathway. BNIP3 and BNIP3L (also known as NIX) are two members of the BH3-only subfamily of Bcl-2 family proteins. After cellular stress, such as hypoxia and nutrient deprivation, BNIP3 and BNIP3L form stable homo-dimerization complexes that co-localize to the outer membrane of the mitochondria. As such, these proteins play important roles in tissue differentiation and the stress responses [4]. It has been described that over-expression of BNIP3 and BNIP3L occurs in various human solid cancers at early stages, as the tumours become hypoxic, including breast cancers [5].

Cancer stem cells (CSCs) are a small sub-population of cells with stem cell-like features, such as self-renewal, tumour initiation capability, high proliferation rates and/or drug-resistance. CSCs are responsible for cancer recurrence, treatment failure, and metastatic dissemination. Accordingly, the elimination of CSCs represents one of the most important new therapeutic approaches in cancer treatment [6]. Mitophagy has been implicated in many aspects of the CSC phenotype, such as self-renewal, cell propagation, and tumorigenic ability [7]. Mitophagy sustains CSCs under adverse conditions or confers chemo-resistance [8]. For those reasons, mitophagy could be a promising target for CSC eradication [9].

In the present study, we have used a BNIP3(L)-promoter-eGFP-reporter system to identify and purify mitophagy-high sub-population(s) of MCF7 and MDA-MB-231 breast cancer cells. After fractionation of these cells by flow cytometry (FACS), we have studied the role of mitophagy in CSCs, demonstrating that this type of mitochondrial autophagy enhances CSC anchorage-independent propagation (i.e., mammosphere formation), and correlates with higher metabolic activity, ATP production, and anti-oxidative capacity, as well as increased proliferation and cell migration.

## RESULTS

### The BNIP3(L)-eGFP reporter system permits the enrichment of a sub-population of cells, with higher levels of lysosomal mass and enhanced basal mitophagy activity

Here, we developed a new model system to enrich sub-populations of cancer cells with higher levels of basal mitophagy, to study the role of mitophagy in cancer stem cell metabolism. More specifically, we enriched MCF7 and MDA-MB-231 sub-populations with higher mitophagy levels, based on the expression of two proteins mechanistically involved in mitophagy, namely BNIP3 and BNIP3L. Using this reporter system, their high transcriptional activity was linked to the recombinant expression of eGFP, allowing the detection of different fluorescent cell sub-populations by flow cytometry.

**Figure 1:**
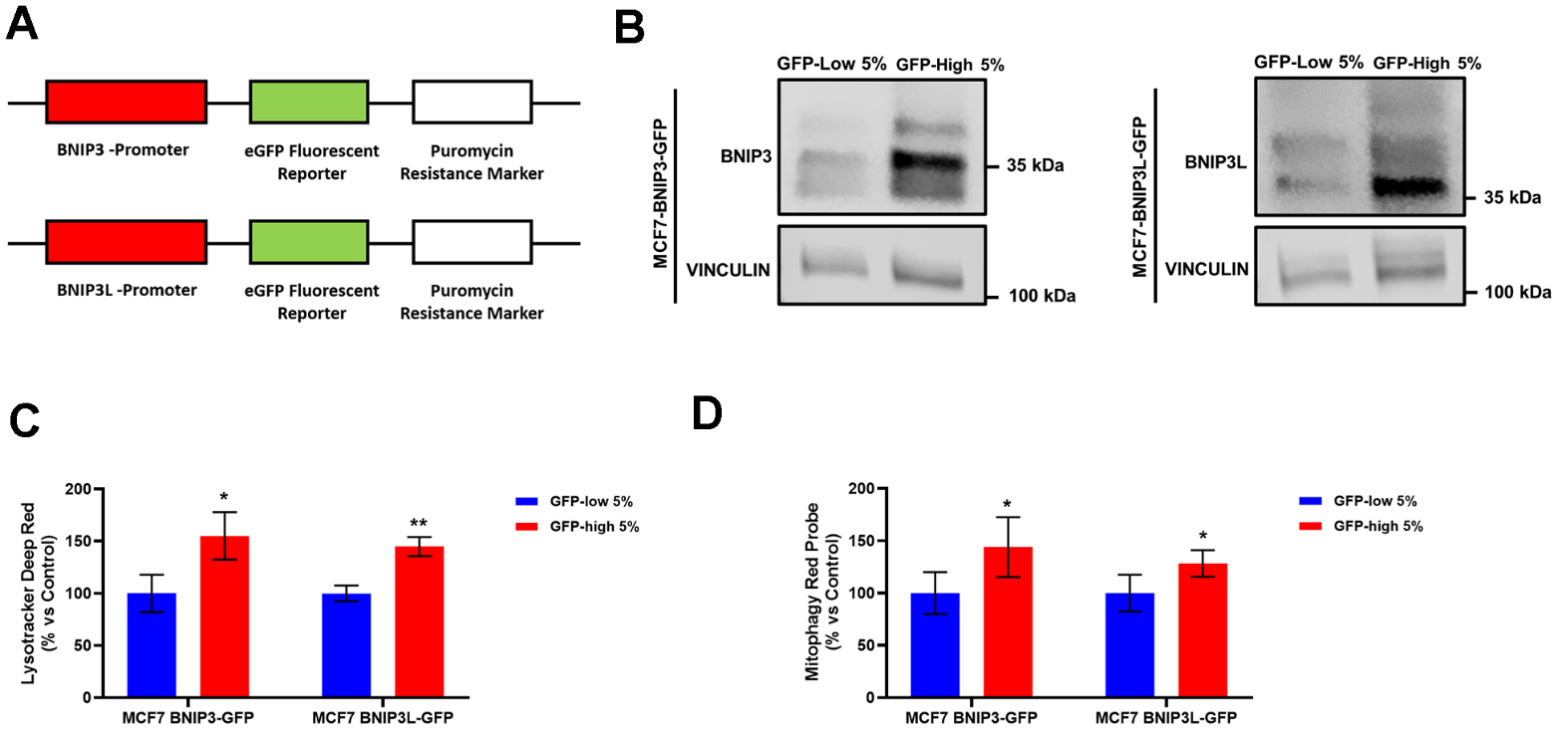
**Generation of MCF7 cells containing a BNIP3(L)-eGFP reporter, permits the selection of a sub-population of cells with high mitophagy activity, which show increased levels of lysosomes and basal mitophagy.** (**A**) Diagram of BNIP3-eGFP-Puro^R^ and BNIP3L-eGFP-Puro^R^ constructs. MCF7 cells stably-transduced with the BNIP3(L)-GFP constructs were subjected to FACS sorting, to isolate the 5% highest GFP (GFP-high) and the 5% lowest GFP (GFP-low) sub-populations. (**B**) BNIP3 and BNIP3L protein levels in GFP-high and GFP-low sub-populations were assessed by Western blotting. Vinculin was used as a protein loading control. (**C**) Lysosomal mass was measured using Lysotracker Deep Red by flow cytometry. Data are shown as the mean ± standard deviation (SD) (n = 3). Statistical significance was determined using an unpaired Student’s t-test, * p ≤ 0.05, ** p ≤ 0.01. (**D**) Mitophagy levels were assessed using the Mitophagy Red Probe by flow cytometry. Data are shown as the mean ± standard deviation (SD) (n = 4). Statistical significance was determined using an unpaired Student’s t-test, * p ≤ 0.05.

Briefly, MCF7 and MDA-MB-231 cells were transduced with two lentiviral constructs, each driving eGFP protein expression, under the control of BNIP3 or BNIP3L promoters, respectively. These DNA constructs also contain a puromycin-resistance cassette, allowing their selection using antibiotic resistance (Figure 1A). Afterwards, stably-transduced MCF7 and MDA-MB-231 cancer cell lines were subjected to flow cytometry to isolate the 5% highest GFP (GFP-high) and the 5% lowest GFP (GFP-low) sub-populations. In this manner, the GFP-high cells represent BNIP3(L)-high transcription levels, and potentially a cell sub-population with higher levels of mitophagy. The GFP-low cells represent the BNIP3(L)-low transcription cell population, a population which serves as a valuable internal control for phenotypic comparisons.

We validated the model by checking the endogenous protein levels of BNIP3 and BNIP3L by Western blot analysis in the GFP-high and GFP-low sub-populations of MCF7-BNIP3-GFP cells and MCF7-BNIP3L-GFP cells. As expected, Figure 1B shows an increase in the protein levels of these two proteins in the GFP-high sub-populations.

In further functional support for an increased mitophagy phenotype, their lysosomal mass (Figure 1C) and mitophagy activity levels (Figure 1D) were both increased in the GFP-high MCF7 cell sub-population, as compared to the GFP-low cells, in both cell lines, MCF7-BNIP3-GFP and MCF7-BNIP3L-GFP. These parameters were tested by flow cytometry after incubation with Lysotracker Deep Red and a mitophagy-specific probe. Taken together, these data directly demonstrate the validity of the model, which allows the enrichment of cancer cells with functionally higher levels of basal mitophagy.

To directly visualize mitophagy and further confirm the validity of our new model, we also used the mitochondrial-targeted red fluorescent protein (RFP), mt-Keima. For this purpose, we sequentially transfected MCF7-BNIP3-GFP and MCF7-BNIP3L-GFP cells with a mt-Keima lenti-viral construct, which contains a hygromycin resistance cassette, since the transfectants were already puromycin-resistant. Thus, we derived two new cell lines: 1) MCF7 cells harboring both BNIP3-GFP and mt-Keima-RFP, as well as 2) MCF7 cells harboring both BNIP3L-GFP and mt-Keima-RFP (Supplementary Figure 1). mt-Keima exhibits pH-dependent excitation at either 458 nm or 561 nm and an emission spectrum that peaks at 620 nm. Importantly, mt-Keima is a red-fluorescent protein (RFP) that is targeted first to mitochondria, via an N-terminal mitochondrial targeting signal, and subsequently only enters lysosomes, during mitophagy. Under these acidic conditions, upon conversion from an autophagosome to an autolysosome, ionized mt-Keima is detected as a red fluorescent signal (with excitation at 561 nm) [10].

**Figure 2:**
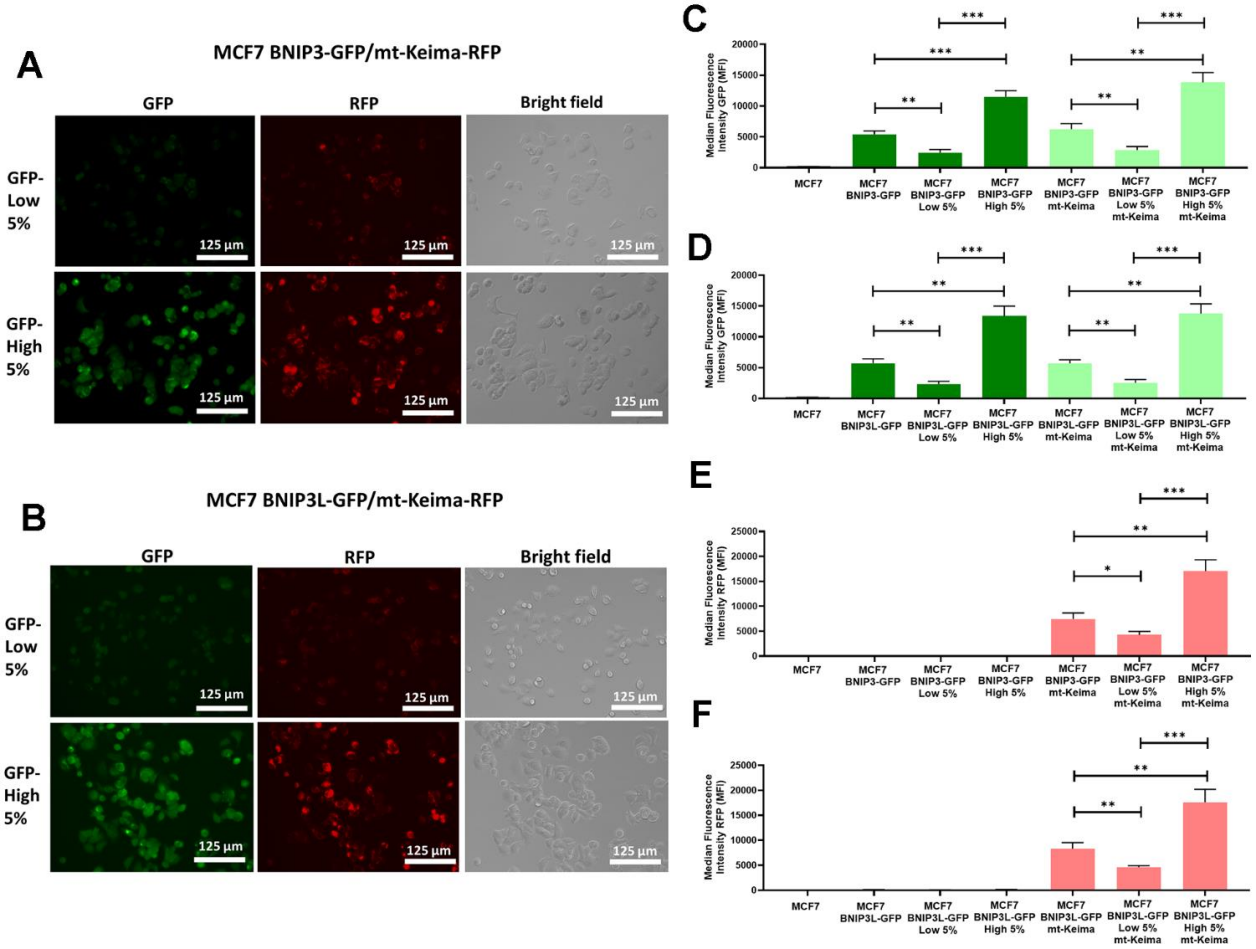
**Assessment of basal mitophagy, using a mitochondrially-targeted red fluorescent protein (mt-Keima-RFP), in doubly-transfected MCF7-BNIP3-GFP and MCF7-BNIP3L-GFP cells.** MCF7 cells stably-transduced with BNIP3(L)-GFP constructs were subjected to a second round of transfections, with a mt-Keima-Hygro^R^ construct to obtain two new cell lines cells, as follows: 1) MCF7 BNIP3-GFP/mt-Keima-RFP and 2) MCF7 BNIP3L-GFP/mt-Keima-RFP. Then, both cell lines were subjected to flow cytometry and sorted according their GFP levels, to isolate the 5% highest GFP (GFP-high) and the 5% lowest GFP (GFP-low) cell sub-populations. (**A**) Representative microscopic images of MCF7 BNIP3-GFP/mt-Keima-RFP cells, after sorting are shown. (**B**) Representative microscopic images of MCF7 BNIP3L-GFP/mt-Keima-RFP cells, after sorting are shown. Note that the green fluorescent signal from BNIP3(L)-GFP very tightly co-segregates visually with the red fluorescent signal from mt-Keima-RFP, an established pH-dependent marker of mitophagy. Corresponding quantitative analysis of this co-segregation by FACS is shown in panels (**C**–**F**) including various other controls, such as single transfectants and untransfected MCF7 cells. (**C**) GFP levels in MCF7 BNIP3-GFP cells and MCF7 BNIP3-GFP/mt-Keima-RFP cells after sorting for the 5% highest GFP (GFP-high) and the 5% lowest GFP (GFP-low) sub-populations. (**D**) GFP levels in MCF7 BNIP3L-GFP cells and MCF7 BNIP3L-GFP/mt-Keima-RFP cells after sorting for the 5% highest GFP (GFP-high) and the 5% lowest GFP (GFP-low) sub-populations. (**E**) RFP levels in MCF7 BNIP3-GFP cells and MCF7 BNIP3-GFP/mt-Keima-RFP cells after sorting for the 5% highest GFP (GFP-high) and the 5% lowest GFP (GFP-low) sub-populations. (**F**) RFP levels in MCF7 BNIP3L-GFP cells and MCF7 BNIP3L-GFP/mt-Keima-RFP cells after sorting for the 5% highest GFP (GFP-high) and the 5% lowest GFP (GFP-low) sub-populations. Note that the BNIP3(L)-GFP green signal quantitatively co-segregates with the red signal from mt-Keima-RFP, which is a well-established red fluorescent marker of mitochondria that are being digested within the low-pH/acidic micro-environment of the auto-lysosome/mito-lysosome. Data are shown as the mean ± standard deviation (SD) (n = 3). Statistical significance was determined using an unpaired Student’s t-test, * p ≤ 0.05, ** p ≤ 0.01, *** p ≤ 0.001.

Firstly, we sorted the two cells lines based on the GFP levels collecting the 5% highest GFP (GFP-high) and the 5% lowest GFP (GFP-low) sub-populations and checked the green and red fluorescence levels by microscopy. As predicted, both green and red fluorescence levels are increased in the 5% highest GFP sub-populations in both MCF7 BNIP3-GFP/mt-Keima-RFP cells (Figure 2A) and MCF7 BNIP3L-GFP/mt-Keima-RFP cells (Figure 2B).

We also quantitatively confirmed these observations by FACS analysis. For this purpose, we used the BL1 channel (ex. 488nm, em. 530nm) and YL2 (ex. 561nm, em. 620nm) to monitor green levels and red fluorescence levels, respectively. Figure 2C shows the GFP levels in MCF7, MCF7 BNIP3-GFP cells and MCF7 BNIP3-GFP/mt-Keima-RFP cells. Note that GFP levels are increased in the 5% highest GFP (GFP-high) cells compared with 5% lowest GFP cells in both cell lines, demonstrating that the mt-Keima is not affecting BNIP3-GFP expression levels.

Similarly, we observed nearly identical results in both MCF7 BNIP3L-GFP cells and MCF7 BNIP3L-GFP/mt-Keima-RFP cells (Figure 2D). In contrast, we only observed red signal in MCF7 BNIP3-GFP/mt-Keima-RFP cells and MCF7 BNIP3L-GFP/mt-Keima-RFP cells in the YL2 channel, and that signal was significantly higher in the 5% highest GFP (GFP-high) (Figure 2E, 2F). We also used untransfected MCF7 cells as a control for all the FACS experiments. Finally, we also sorted MCF7 BNIP3-GFP/mt-Keima-RFP cells and MCF7 BNIP3L-GFP/mt-Keima-RFP cells based on red fluorescence to obtain the 5% highest RFP (mt-Keima-high 5%) and the 5% lowest RFP (mt-Keima-low 5%) sub-populations. We independently observed the highest levels of mammosphere forming ability (Supplementary Figure 2A) and ATP levels (Supplementary Figure 2B) in mt-Keima-high 5%, as compared with the mt-Keima-low 5% population.

Taken together, these experiments provide solid evidence that these BNIP3-GFP and BNIP3L-GFP reporters can be used successfully to isolate cell sub-populations by flow cytometry, with increased basal levels of mitophagy.

### Higher basal mitophagy levels enhance mammosphere formation and CSC markers

**Figure 3:**
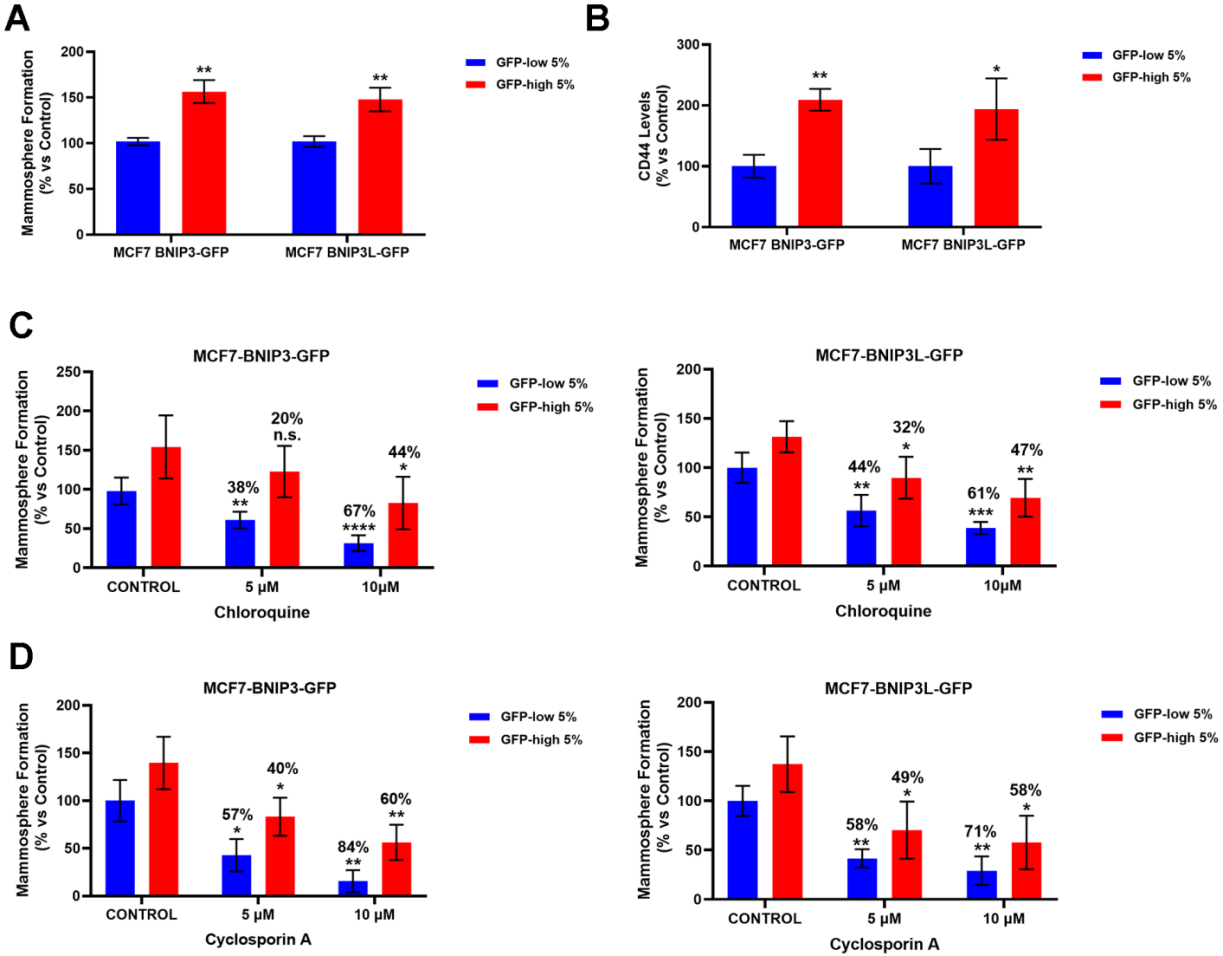
**BNIP3(L)-high MCF7 cells form mammospheres more efficiently and show higher levels of the stemness marker CD44, as well as resistance to treatment with Chloroquine and Cyclosporin A, due to their higher mitophagy levels.** MCF7 cells stably-transduced with the BNIP3(L)-GFP constructs were subjected to FACS sorting, to isolate the 5% highest GFP (GFP-high) and the 5% lowest GFP (GFP-low) sub-populations. (**A**) The GFP-high and GFP-low subpopulations were seeded into low-attachment plates for mammosphere assays and analysed after 5 days. Data are shown as the mean ± standard deviation (SD) (n = 3). Statistical significance was determined using an unpaired Student’s t-test, ** p ≤ 0.01. (**B**) CD44 levels were determined with an APC mouse anti-Human CD44 antibody by flow cytometry. Data are shown as the mean ± standard deviation (SD) (n = 3). Statistical significance was determined using an unpaired Student’s t-test, * p ≤ 0.05, ** p ≤ 0.01. (**C**) Autophagy inhibitor Chloroquine was tested by using the mammosphere assay to determine the differential sensitivity of GFP-high and GFP-low subpopulations to this drug. Both subpopulations were plated in low-attachment plates for mammosphere assays and incubated with Chloroquine at the indicated concentrations. The number of mammospheres were quantitated after 5 days. The percentage indicated at the top of the bars represents the decrease of that bar compared with its own untreated control (GFP-high treated compared to GPF-high untreated, and GFP-low treated compared to GFP-low untreated). Data are shown as the mean ± SD (n = 4). Statistical significance was determined using one-way ANOVA, Dunnett’s multiple comparisons test, *p ≤ 0.05, **p ≤ 0.01, ***p < 0.001, ****p < 0.0001, n.s. not statistically significant. (**D**) To evaluate the differential sensitivity of GFP-high and GFP-low subpopulations to a specific mitophagy inhibitor Cyclosporin A, we used a mammosphere assay. Both subpopulations were plated in low-attachment plates for mammosphere assays and incubated with Cyclosporin A, at the indicated concentrations. The number of mammospheres were quantitated after 5 days. The percentage indicated at the top of the bars represents the decrease of that bar compared with its own untreated control (GFP-high treated compared to GPF-high untreated, and GFP-low treated compared to GFP-low untreated). Data are shown as mean ± SD (n = 3). Statistical significance was determined using one-way ANOVA, Dunnett’s multiple comparisons test, *p ≤ 0.05, **p ≤ 0.01.

Next, we studied the role of the mitophagy in the propagation of cancer stem cells. For this purpose, we compared the mammosphere-forming ability between BNIP3(L) GFP-high and BNIP3(L) GFP-low MCF7 cells. GFP-high cells showed a statistically significant increase in mammospheres formation (Figure 3A), which functionally represents anchorage-independent CSC propagation, under 3D conditions. Furthermore, the GFP-high cells showed significantly higher levels of CD44 (Figure 3B), a well-known cell surface marker of CSCs.

In order to further validate that the results obtained in the mammosphere assay were due to mitophagy, we treated the GFP-high and GFP-low MCF7 cells with Chloroquine, one of the inhibitors most frequently used to block autophagy, and Cyclosporin A, a specific inhibitor of mitophagy. For this purpose, we used the mammosphere assay as a functional read-out; cells were seeded under non-adherent condition and treated with increasing concentrations of the drugs, as indicated, for 5 days.

Note that, in both cell lines (MCF7-BNIP3-GFP and MCF7-BNIP3L-GFP), the GFP-low subpopulations were clearly more sensitive to Chloroquine (Figure 3C) and Cyclosporin A treatment (Figure 3D), as compared with the GFP-high cells. This consistently occurred at every concentration tested.

In both treatments, mammosphere formation by GFP-high cells was decreased by at least 10% less (even 20% in some treatments) than the GFP-low cells, suggesting that the high levels of endogenous mitophagy in the GFP-high cells made these cells more resistant to the autophagy and mitophagy inhibitors, further demonstrating that mitophagy is functionally implicated in mammosphere formation.

### Mitophagy-high cell sub-populations are more metabolically active and show an increase in mitochondrial activity, as well as a drug-resistance phenotype (Tamoxifen/Palbociclib)

**Figure 4:**
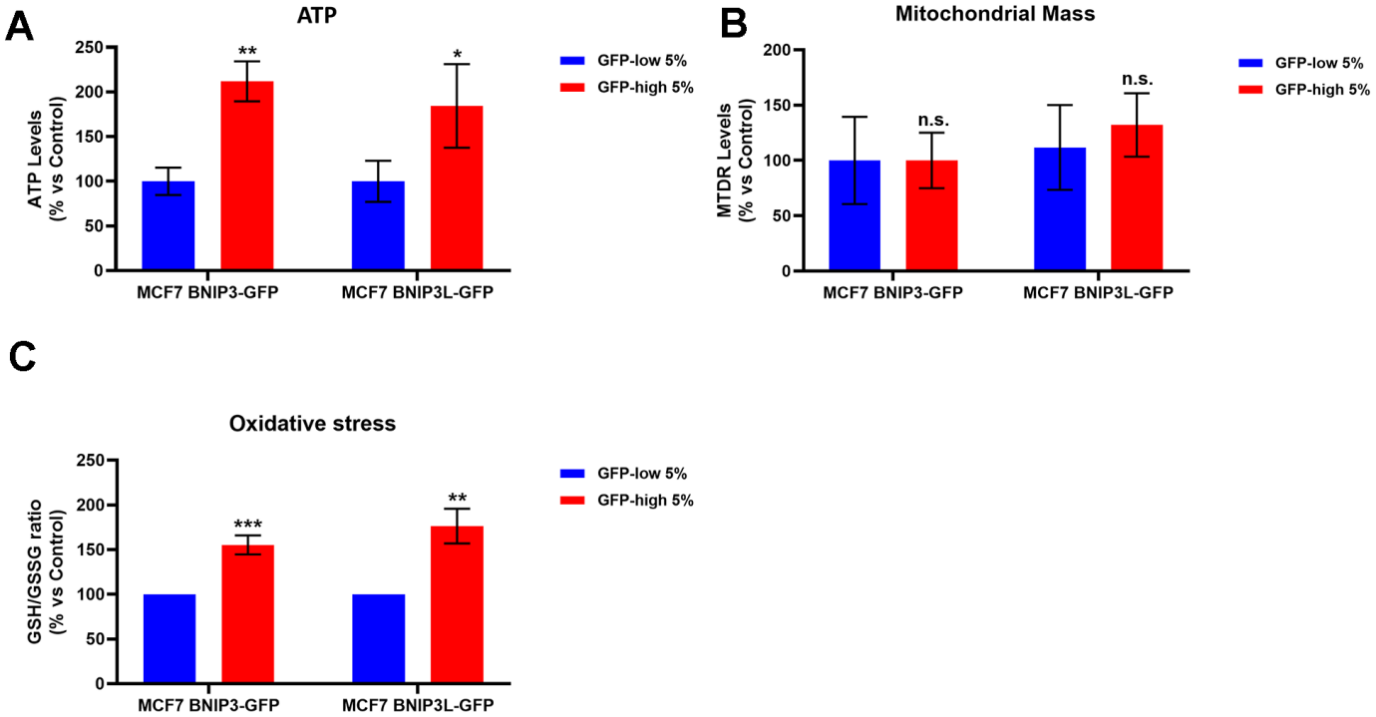
**BNIP3(L)-high MCF7 cells show higher ATP levels and higher anti-oxidant capacity; however, mitochondrial mass remains unchanged.** MCF7 cells stably-transduced with the BNIP3(L)-GFP constructs were subjected to FACS sorting, to isolate the 5% highest GFP (GFP-high) and the 5% lowest GFP (GFP-low) sub-populations. (**A**) The GFP-high and GFP-low subpopulations were plated in complete DMEM medium and incubated with the Cell-Titer-Glo 2.0 Reagent for 15 min to determinate the ATP levels. Data are shown as the mean ± standard deviation (SD) (n = 3). Statistical significance was determined using an unpaired Student’s t-test, * p ≤ 0.05, ** p ≤ 0.01. (**B**) Mitochondrial mass was assessed using MitoTracker Deep Red by flow cytometry. Data are shown as mean ± standard deviation (SD) (n = 3). Statistical significance was determined using an unpaired Student’s t-test, n.s. not statistically significant. (**C**) The anti-oxidant capacity was measured with the GSH/GSSG-Glo Assay. Data are shown as the mean ± standard deviation (SD) (n = 3). Statistical significance was determined using an unpaired Student’s t-test, ** p ≤ 0.01, ***p ≤ 0.001.

To better understand the effects of mitophagy on cancer stem cells, we analysed their metabolism and mitochondrial status. We first observed a significant increase in the ATP levels in the GFP-high cells, as compared with the GFP-low cells (Figure 4A) in both cell lines, MCF7-BNIP3-GFP and MCF7-BNIP3L-GFP. However, mitochondrial mass, as measured using a well-established fluorescent probe, namely MitoTraker Deep-Red, showed similar values between these 2 populations (Figure 4B).

In addition, the GSH/GSSG ratio showed a significant increase in the GFP-high cells (Figure 4C). These results indicated that cells with high levels of mitophagy (GFP-high cells) showed better mitochondrial function, since cells with the same levels of mitochondrial mass, as compared with the control subpopulation (GFP-low cells), exhibited elevated levels of both ATP and anti-oxidant capacity.

**Figure 5:**
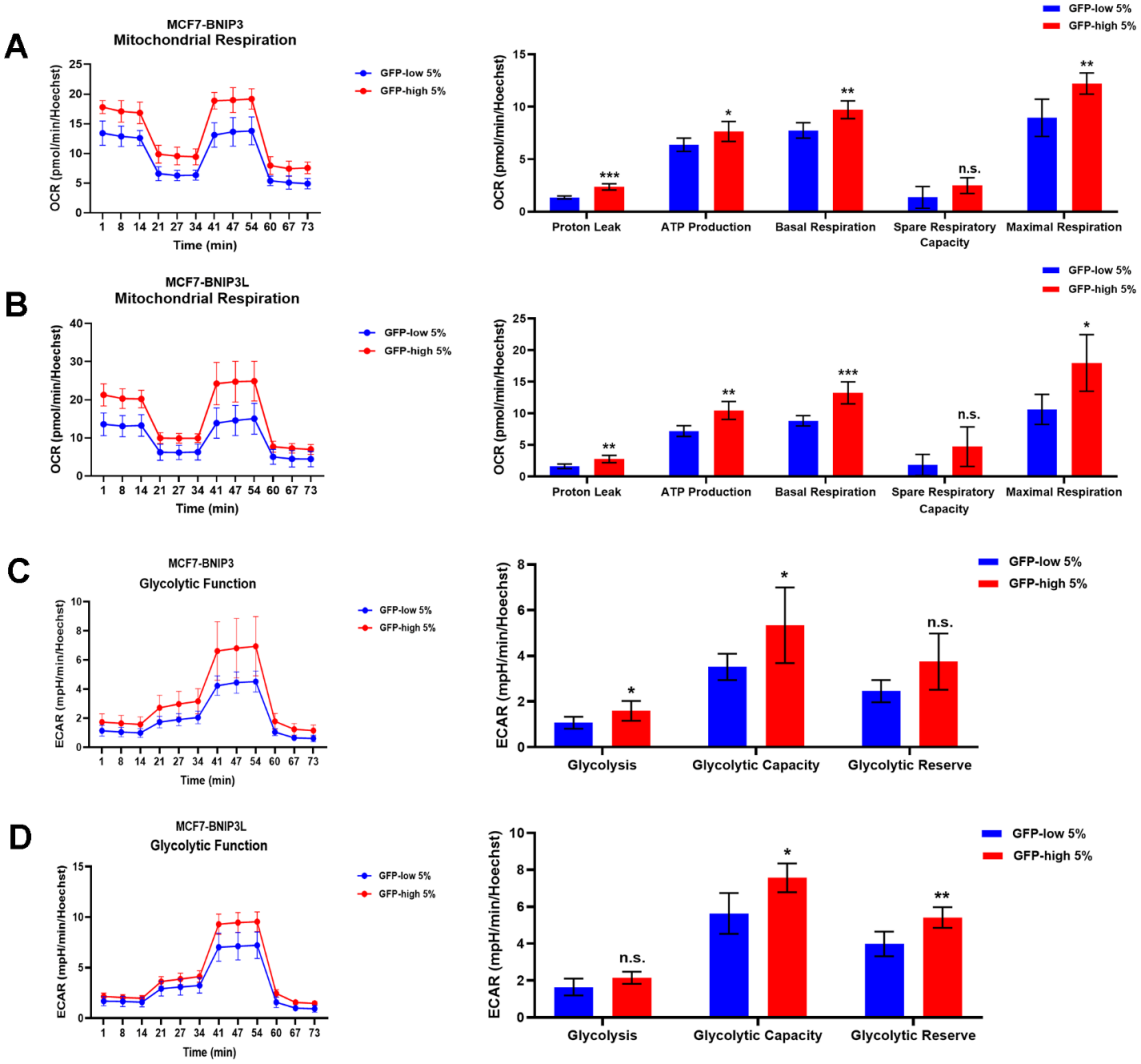
**Mitochondrial respiration and glycolysis are significantly enhanced in BNIP3(L)-high MCF7 cells.** MCF7 cells stably-transduced with the BNIP3(L)-GFP constructs were subjected to FACS sorting, to isolate the 5% highest GFP (GFP-high) and the 5% lowest GFP (GFP-low) sub-populations. The Seahorse XF96 analyser was employed to assess their mitochondrial and glycolytic function. Mitochondrial function results for BNIP3-GFP cells (**A**) and BNIP3L-GFP (**B**) are shown as an OCR tracing in left panel, and bar graphs for proton leak, ATP production, basal respiration, spare respiratory capacity and maximal respiration, obtained from the OCR quantification. Data are shown as the mean ± standard deviation (SD) (n = 5). Statistical significance was determined using an unpaired Student’s t-test, * p ≤ 0.05, ** p ≤ 0.01, *** p ≤ 0.001, n.s. not statistically significant. Glycolytic function results for BNIP3-GFP cells (**C**) and BNIP3L-GFP (**D**) are shown as an ECAR tracing in left panel, and bar graphs for glycolysis, glycolytic capacity and glycolytic reserve, obtained from the ECAR quantification. Data are shown as the mean ± standard deviation (SD) (n = 5). Statistical significance was determined using an unpaired Student’s t-test, * p ≤ 0.05, ** p ≤ 0.01, n.s. not statistically significant.

**Figure 6:**
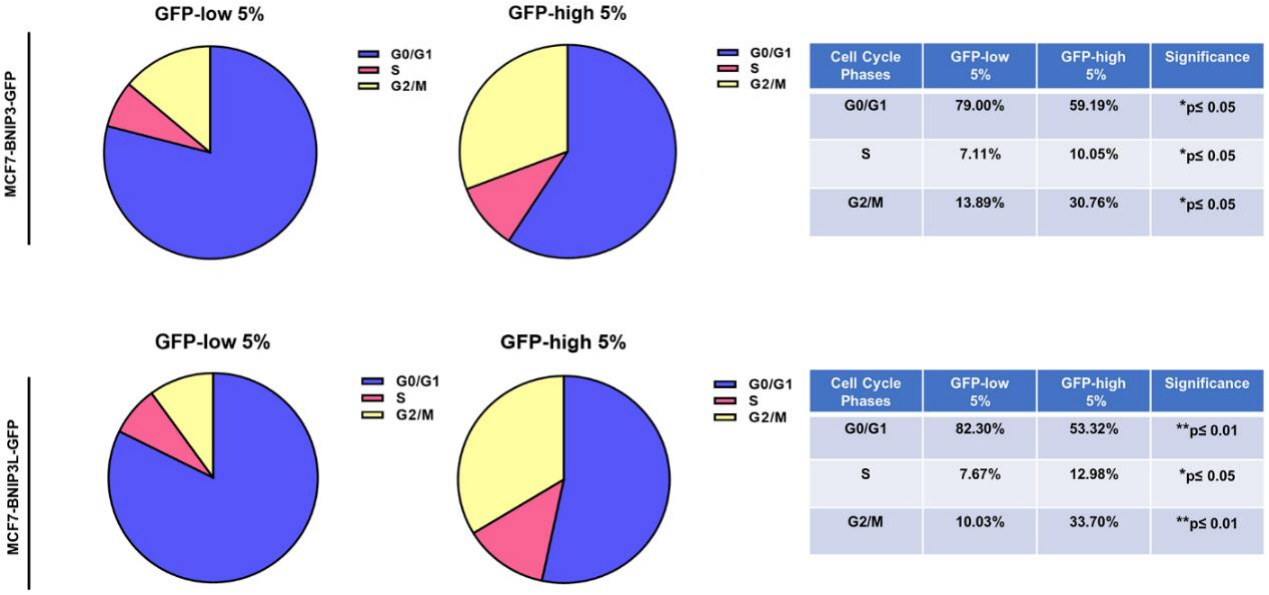
**Cell cycle progression is elevated in BNIP3(L)-high MCF7 cells.** MCF7 cells stably-transduced with the BNIP3(L)-GFP constructs were subjected to FACS sorting, to isolate the 5% highest GFP (GFP-high) and the 5% lowest GFP (GFP-low) sub-populations. Cell cycle progression was evaluated with propidium iodide by flow cytometry. The percentage of cells in G0/G1, S, and G2/M phases of the cell cycle are represented in the pie graphs. Data are shown as the mean ± standard deviation (SD) (n = 3). Statistical significance was determined using an unpaired Student’s t-test, *p ≤ 0.05, **p ≤ 0.01.

Using the Seahorse XFe96 bioenergetic analyser, Figure 5 clearly demonstrates that GFP-high cells were more metabolically active than GFP-low cells and showed a significant increase in their mitochondrial respiration and glycolytic function in the GFP-high cells in both cell lines, MCF7-BNIP3-GFP and MCF7-BNIP3L-GFP. Furthermore, GFP-high cells were more proliferative, as we observed by cell cycle analysis (Figure 6) that these cells showed a significant decrease in the G0/G1-phase, with corresponding increases in S-phase and the G2/M-phase.

**Figure 7:**
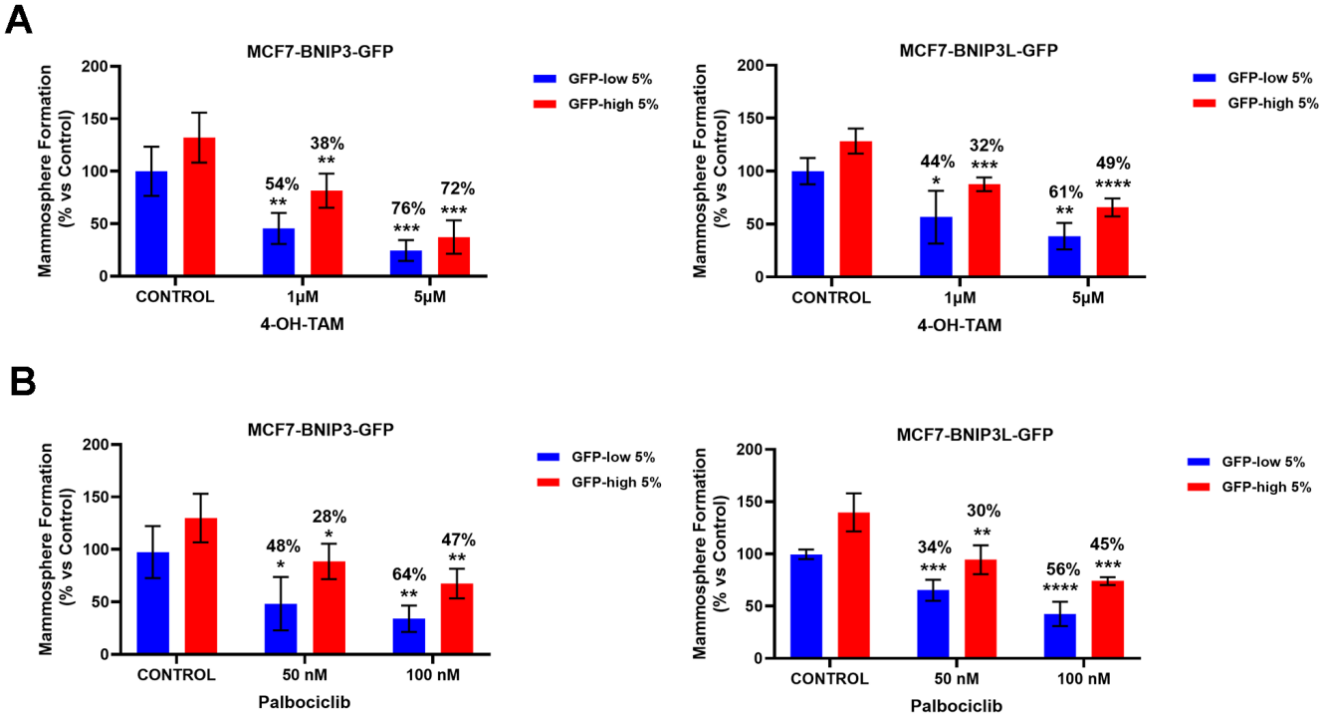
**BNIP3(L)-high MCF7 cells show multi-drug resistance to treatment with 4-OH-Tamoxifen and Palbociclib.** MCF7 cells stably-transduced with the BNIP3(L)-GFP constructs were subjected to FACS sorting, to isolate the 5% highest GFP (GFP-high) and the 5% lowest GFP (GFP-low) sub-populations. Differential sensitivity of GFP-high and GFP-low subpopulations to 4-OH-Tamoxifen (**A**) and Palbociclib (**B**) was evaluated by using the mammosphere assay. The GFP-high and GFP-low subpopulations were plated in low-attachment plates for mammosphere assays and incubated with 4-OH-Tamoxifen or Palbociclib, at the indicated concentrations. The number of mammospheres were quantitated after 5 days. The percentage indicated at the top of the bars represents the decrease of that bar compared with its own untreated control (GFP-high treated compared to GPF-high untreated, and GFP-low treated compared to GFP-low untreated). Data are shown as the mean ± SD (n = 4). Statistical significance was determined using one-way ANOVA, Dunnett’s multiple comparisons test, *p ≤ 0.05, **p ≤ 0.01, ***p < 0.001.

In Figure 7, we evaluated the possible drug resistant phenotype(s) of the GFP-high and GFP-low cell sub-populations to 4-OH-Tamoxifen, an FDA-approved drug used in ER(+) breast cancer cells treatment, and Palbociclib, a CDK4/6 inhibitor. As performed previously with the autophagy and mitophagy inhibitors, we used a mammosphere assay to functionally evaluate their sensitivity/resistance. Briefly, the cells were seeded under non-adherent conditions and treated with different concentrations of the drugs, for a period of 5 days.

Interestingly, mammosphere formation by GFP-low cell sub-populations was more sensitive to Tamoxifen treatment (Figure 7A), in both MCF7-BNIP3-GFP and MCF7-BNIP3L-GFP cells. However, mammosphere formation by BNIP3(L)-high cells, showed that they were clearly more resistant to Tamoxifen treatment, as compared with vehicle-treated control cells. Experiments with Palbociclib (Figure 7B) also showed exactly the same trends, with BNIP3(L)-high cells demonstrating that they were clearly more resistant to Palbociclib treatment, as compared with vehicle-treated control cells.

### BNIP3(L)-High MDA-MB-231 cells show significant increases in basal mitophagy, anchorage-independent growth, ATP production and cell migration

Metastatic capacity is an important feature of the CSC phenotype. Thus, we examined the migration levels of the GFP-high and GFP-low cell sub-populations in the MDA-MB-231-BNIP3-GFP and MDA-MB-231-BNIP3L-GFP cell lines, obtained with the same protocol that was followed for the MCF7 cells, to separate the two sub-populations.

**Figure 8:**
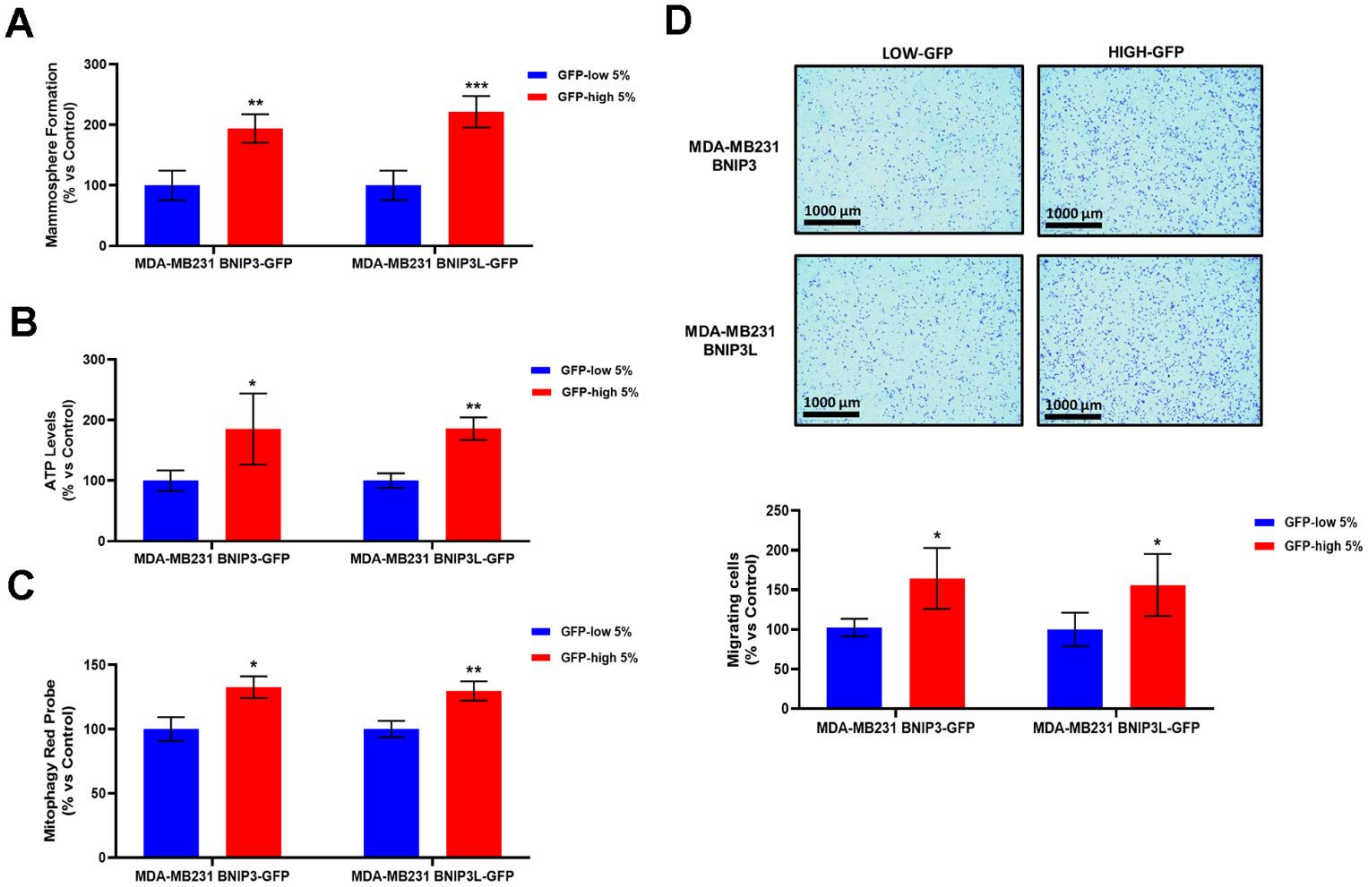
**BNIP3(L)-high MDA-MB-231 cells form mammospheres more efficiently, produce more ATP, and show increased levels of basal mitophagy, as well as enhanced cell migration.** MDA-MB-231 cells stably-transduced with the BNIP3(L)-GFP constructs were subjected to FACS sorting, to isolate the 5% highest GFP (GFP-high) and the 5% lowest GFP (GFP-low) sub-populations. (**A**) The GFP-high and GFP-low subpopulations were seeded in low-attachment plates for mammosphere assays and analysed after 5 days. Data are shown as the mean ± standard deviation (SD) (n = 4). Statistical significance was determined using an unpaired Student’s t-test, ** p ≤ 0.01, ***p ≤ 0.001. (**B**) The GFP-high and GFP-low subpopulations were plated in complete DMEM medium and incubated with the Cell-Titer-Glo 2.0 Reagent for 15 min to determinate the ATP levels. Data are shown as the mean ± standard deviation (SD) (n = 4). Statistical significance was determined using an unpaired Student’s t-test, * p ≤ 0.05, **p ≤ 0.01. (**C**) Mitophagy levels were assessed with the Mitophagy Red Probe, by flow cytometry. Data are shown as mean ± standard deviation (SD) (n = 3). Statistical significance was determined using an unpaired Student’s t-test, *p ≤ 0.05, **p ≤ 0.01. (**D**) The migratory capacity of the two sub-populations was assessed using Transwells (24 well-inserts, with an uncoated PET membrane). The cells were allowed to migrate across an uncoated membrane (with 8μm pores) for 16 hours. In the upper panel, the images show the migration for a representative experiment. In the lower panel, the bars show the quantification of the migration. Data are shown as the mean ± standard deviation (SD) (n = 4). Statistical significance was determined using an unpaired Student’s t-test, * p ≤ 0.05.

We used MDA-MB-231 cells as they are a well-established model for the study of cell motility and metastasis. As a first step, GFP-high MDA-MB-231 cells showed a stemness phenotype since they had increased mammosphere capacity (Figure 8A) and higher ATP levels (Figure 8B), as we observed previously in the GFP-high MCF7 cells. Furthermore, GFP-high MDA-MB-231 cells showed higher levels of mitophagy, as measured using a mitophagy-specific probe (Figure 8C), as used previously in MCF7 cells (Figure 1D).

Finally, as predicted, GFP-high MDA-MB-231 cells exhibited higher levels of cell migration (Figure 8D), revealing that higher levels of mitophagy indeed contribute to both more aggressive phenotypes, namely stemness and cell motility.

## DISCUSSION

CSCs are responsible for cancer relapse, therapy-resistance, and metastatic dissemination. Therefore, CSC elimination is necessary to prevent cancer recurrence and improve long-term patient outcomes. The search of new targets against CSCs is essential for the success of cancer treatment [11].

One of these new approaches may be the targeting of mitophagy, a selective form of macro-autophagy, in which dysfunctional mitochondria are degraded in auto-phago-lysosomes, as this mechanism in CSCs has been widely reported. For example, mitophagy in oesophageal squamous cell carcinoma prevents cell death and promotes expression of CD44 (a CSC marker) [12], maintains hepatic CSCs by eliminating mitochondrial p53 [13], and plays a key role in maintaining of self-renewal in leukaemia stem cells [14].

Many pathways have been reported to regulate mitophagy, but we focused on BNIP3/BNIP3L-dependent mitophagy, as these two proapoptotic BH3-only proteins are rapidly induced at a transcriptional level by mitochondrial membrane stress and they interact with processed LC3/GABARAP to promote mitophagy [1].

Here, we present a new genetic-based fluorescence approach to isolate mitophagy-high cell sub-populations based on the differential expression levels of BNIP3 or BNIP3L, in either MCF7 or MDA-MB-231 cell model systems. For this purpose, we used the promoter of BNIP3/BNIP3L proteins linked to an eGFP-based reporter system. This allowed us to isolate two distinct sub-populations of cells, according to their levels of eGFP fluorescence. GFP-high cells corresponded to high BNIP3(L) activity levels and represented a mitophagy-high enriched cell sub-population. In contrast, GFP-low cells correspond to a mitophagy-low sub-population, which serves as an internal control and comparative reference point.

To validate the model, we demonstrated that the high transcriptional levels of BNIP3/BNIP3L activity correspond to higher expression levels of BNIP3/BNIP3L protein by immunoblot analysis. In addition, we measured lysosomal mass by FACS analysis, an important component in the mitophagy mechanism. Lysosomal mass was significantly increased in the mitophagy-high sub-population, as would be expected as they are an essential part of the mitophagy process [2, 15].

Additionally, we directly assessed the mitophagy levels with a specific probe by FACS analysis. As predicted, mitophagy activity was functionally increased in the GFP-high cells in both cell lines, MCF7-BNIP3-GFP and MCF7-BNIP3L-GFP, specifically validating that high BNIP3(L) transcription levels are directly correlated with higher mitophagy levels.

To further validate the new model, we used the mitochondrially-targeted red fluorescent protein (RFP), namely mt-Keima. This probe has been widely-used for the study of mitophagy and is based on the pH differences occurring during the stages of mitophagy [10, 16, 17]. Double transfection of cells with BNIP3(L)-GFP and mt-Keima permitted us to confirm the increased levels of basal mitophagy in BNIP3(L) GFP-high cells and the increased levels of ATP and mammosphere formation related with mitophagy.

Moreover, we observed that BNIP3(L)-high MCF7 cells showed a significant increase in their mammosphere forming ability, as well as in their expression levels of a well-known epithelial stem cell marker, namely CD44, indicating that this sub-population is functionally enriched in CSC activity. In further support of the idea that mammosphere forming activity is mitophagy-dependent, mitophagy-high cells were more resistant to two known mitophagy inhibitors, namely Chloroquine and Cyclosporin A. Chloroquine inhibits autophagy by increasing lysosomal pH, preventing proper lysosomal function [15, 18]. In addition, cyclosporin A is a specific inhibitor of mitophagy. Cyclosporin A maintains mitochondrial membrane potential by inhibiting Cyclophilin D (CypD), thereby preventing mitophagy [19, 20].

We further validated this approach, by analysing other CSCs features. Firstly, we detected increased levels of ATP production and anti-oxidant capacity in BNIP3(L)-high MCF7 cells. Remarkably, the mitochondrial mass remained unchanged, as compared with control cells BNIP3(L)-low MCF7. Many studies have previously described a decrease in mitochondrial mass, due to mitophagy [20, 21]. However, in our model system, this decrease did not occur; instead, we observed better overall mitochondrial function. Metabolic flux analysis was also conducted using the Seahorse XFe96, to quantitatively measure oxidative mitochondrial function and glycolytic activity. We reported a significant increase in both mitochondrial and glycolytic function in BNIP3(L)-high MCF7 cells. Finally, BNIP3(L)-high MCF7 cells were more proliferative, showing increases in the S-phase and the G2/M-phase of the cell cycle, confirming the presence of a proliferative and energetic CSC phenotype, with increased mitochondrial ATP production [22].

Similarly, drug resistance is another feature of the CSC phenotype. Chemo- and radio-resistance in CSCs has been reported in several studies [23]. In accordance with these reported observations, BNIP3(L)-high MCF7 cells also demonstrated drug resistance to Tamoxifen, a FDA-approved drug used in ER(+) breast cancer treatment and Palbociclib, a CDK4/6 inhibitor. Therapy fails clinically in many cases, due an existing or by developing a drug resistant sub-population of cancer cells, producing tumour recurrence and metastasis.

CSCs have also been implicated in metastatic dissemination. To study this process further, we used MDA-MB-231 cells, a well-established model for the study of cell motility. Remarkably, BNIP3(L)-high MDA-MB-231 cells showed increases in their capacity to undergo cell migration. We also examined their mammosphere formation ability and their energetic status, confirming that BNIP3(L)-high MDA-MB-231 cells possess a stem-like and hyper-energetic phenotype. Moreover, we validated that BNIP3(L)-high MDA-MB-231 cells demonstrated higher levels of mitophagy. In summary, our current work has provided a novel strategy to enrich for a sub-population of cancer cells, with high basal levels of mitophagy.

## MATERIALS AND METHODS

### Cell models and other reagents

MCF7 and MDA-MB-231 cells were obtained commercially from the American Type Culture Collection (ATCC). Cells were maintained in Dulbecco’s Modified Eagle Medium (DMEM; Sigma-Aldrich, #D6546) supplemented with 10% Fetal Bovine Serum (HI FBS; Gibco, #10082-147), 2mM GlutaMAX (Gibco, #35050-061), and 1% Penicillin-Streptomycin (Sigma-Aldrich, #P0781). Cells were grown at 37° C in a 5% CO_2_ humidified incubator. 4-OH-Tamoxifen, Palbociclib, Chloroquine phosphate and Cyclosporin A, were all purchased from Sigma-Aldrich (catalogue numbers: #579002, #PZ0383, #PHR1258, #30024, respectively).

### Viral transduction and cell selection

Lentiviral constructs from GeneCopoeia (BNIP3-eGFP-Puro^R^, #HPRM45275-LvPF02; BNIP3L-eGFP-Puro^R^, #HPRM45442- LvPF02) were amplified and used to stably-transduce MCF7 and MDA-MB-231 cells. After cell transduction, the cell lines were selected with puromycin for 7-10 days. MCF7 and MDA-MB-231 cells stably transduced with the different constructs were sorted using the SONY SH800 Cell Sorter, and the 5% highest (GFP-high) and the 5% lowest (GFP-low) sub-populations of cells were isolated. After sorting, these cell sub-populations were subjected to different functional assays, to characterize their phenotypic differences experimentally.

### Mitophagy detection using mt-Keima, a pH-dependent red fluorescent protein (RFP)

As previously described [10, 16], mt-Keima exhibits pH-dependent and bimodal excitation at either 458 nm or 561 nm, with an emission peak at 620nm. Only after autolysosome formation, which results in an acidic micro-environment, ionized mt-Keima is detected as a red fluorescent signal (with excitation at 561 nm). The mt-Keima-Hygro^R^ construct was transduced into MCF7 cells previously transfected with BNIP3-eGFP-Puro^R^ and BNIP3L-eGFP-Puro^R^. Therefore, a sequential double transfection and selection was carried with puromycin and hygromycin, to obtain two cells lines: 1) MCF7 (BNIP3-GFP + mt-Keima-RFP) and 2) MCF7 (BNIP3l-GFP + mt-Keima-RFP).

After 2 weeks of selection, the cells were sorted using the SONY SH800 Cell Sorter, and the 5% highest (GFP-high or RFP-high) and the 5% lowest (GFP-low or RFP-low) sub-populations of cells were isolated. Different functional assays were executed after sorting (Supplementary Figure 1).

The mt-mKeima-RFP sequence from the following DNA construct (https://www.addgene.org/72342/) was used to generate a lentiviral vector, with hygromycin resistance (Hygro^R^) (subcloned into the plasmid pReceiver-Lv152; GeneCopoeia, Inc.).

This DNA coding sequence includes a tandem-repeat of the COX8A mitochondrial-targeting pre-sequence (MSVLTPLLLRGLTGSARRLPVPRAKIHSLPPEGKLG) fused in-frame to mKeima (monomeric Keima). For convenience, the mito-mKeima (894bp) insert reference DNA sequence is shown here:

ATGTCCGTCCTGACGCCGCTGCTGCTGCGGGGCTTGACAGGCTCGGCCCGGCGGCTCCCAGTGCCGCGCGCCAAGATCCATTCGTTGCCGCCGGAGGGGAAGCTTGGGATGTCCGTCCTGACGCCGCTGCTGCTGCGGGGCTTGACAGGCTCGGCCCGGCGGCTCCCAGTGCCGCGCGCCAAGATCCATTCGTTGCCGCCGGAGGGGAAGCTCGGGGGATCCGCCATGGTGAGCGTGATCGCCAAGCAGATGACCTACAAGGTGTACATGAGCGGCACCGTGAACGGCCACTACTTCGAGGTGGAGGGCGACGGCAAGGGCAAGCCCTACGAGGGCGAGCAGACCGTGAAGCTGACCGTGACCAAGGGTGGCCCCCTGCCCTTCGCCTGGGACATCCTGAGCCCCCAGCTCCAGTACGGCAGCATCCCCTTCACCAAGTACCCCGAGGACATCCCCGACTACTTCAAGCAGAGCTTCCCCGAGGGCTACACCTGGGAGCGCAGCATGAACTTCGAGGACGGCGCCGTGTGCACCGTGAGCAACGACAGCAGCATCCAGGGCAACTGCTTCATCTACAACGTGAAGATCAGCGGCGAGAACTTCCCCCCCAACGGCCCCGTGATGCAGAAGAAGACCCAGGGCTGGGAGCCCAGCACCGAGCGCCTGTTCGCCCGCGACGGAATGCTGATCGGCAACGACTACATGGCCCTGAAGCTGGAGGGCGGCGGCCACTACCTGTGCGAGTTCAAGAGCACCTACAAGGCCAAGAAGCCCGTGAGGATGCCCGGCCGCCACGAGATCGACCGCAAGCTGGACGTGACCAGCCACAACCGCGACTACACCAGCGTGGAGCAGTGCGAGATCGCCATCGCCCGCCACTCCCTGCTGGGCTAA.

Similarly, the protein sequence is also shown below, with the tandem COX8A mitochondrial-targeting sequences and the mKeima sequence (https://www.snapgene.com/plasmids/fluorescent_protein_genes_and_plasmids/mKeima-Red).

MSVLTPLLLRGLTGSARRLPVPRAKIHSLPPEGKLGMSVLTPLLLRGLTGSARRLPVPRAKIHSLPPEGKLGGSAMVSVIAKQMTYKVYMSGTVNGHYFEVEGDGKGKPYEGEQTVKLTVTKGGPLPFAWDILSPQLQYGSIPFTKYPEDIPDYFKQSFPEGYTWERSMNFEDGAVCTVSNDSSIQGNCFIYNVKISGENFPPNGPVMQKKTQGWEPSTERLFARDGMLIGNDYMALKLEGGGHYLCEFKSTYKAKKPVRMPGRHEIDRKLDVTSHNRDYTSVEQCEIAIARHSLLG.

### Mammosphere formation assay

A single cell suspension was prepared after sorting, using enzymatic (1x Trypsin-EDTA; Sigma Aldrich, #T3924), and manual disaggregation (25-gauge needle). Then, cells were plated at a density of 500 cells/cm^2^ in mammosphere medium (DMEM F12, Gibco, #21041-025; B27, Gibco, #17504-044; 20ng/ml EGF, Peprotech, #AF-100-15; 1% Pen-Strep) under non-adherent conditions. Culture dishes were previously coated with Poly 2-hydroxyethyl methacrylate (poly-HEMA, Sigma-Aldrich, #P3932). Cells were incubated in a humidified atmosphere at 37° C and 5% CO_2_ for 5 days. After incubation, mammospheres greater than 50-μm were counted using an eye-piece graticule.

### Western blotting

After sorting, cells were lysed in RIPA buffer (Sigma-Aldrich, #R0278), containing protease and phosphatase inhibitors (Roche, #04906845001 #05892970001). After protein quantification using the BCA protein assay kit, total lysates were loaded onto SDS-polyacrylamide gels (SDS-PAGE; Mini-Protean TGX Gel, 4-20%, Bio-Rad, # 4561094). The gels were transferred onto 0.2-μm nitrocellulose membranes (Mini Trans-Blot Turbo Transfer Pack, Bio-Rad, #1704158), using the Trans-Blot Turbo Transfer System (Bio-Rad). Membranes were blocked with 5% BSA PBS-T (PBS 1%; 0.5% Tween 20, Sigma-Aldrich, #) for 1 hour at room temperature in an orbital shaker. Subsequently, the membranes were incubated with primary antibodies in 5% BSA/PBS-T for 12–16 h at 4° C, followed by incubation with secondary antibodies (Cell Signalling Technology; Anti-Rabbit IgG HRP Linked #7074; Anti-Mouse IgG HRP Linked #7076) in 5% BSA/PBS-T for 1 h at room temperature. The membranes were developed with SuperSignal West Pico chemiluminescent substrate (ThermoFisher Scientific, #34580). Antibodies against the following proteins were used: BNIP3(Cell Signaling, #44060), BNIP3L (Cell Signaling, #12396) and vinculin (Santa Cruz Biotechnology, #sc-73614). Vinculin was used as protein loading control. The resulting images were acquired using Odyssey XF (LI-COR).

### Mitochondrial and lysosomal staining

After sorting, cells were stained with MitoTracker Deep Red (Invitrogen, #M22426) or Lysotracker Deep Red (Invitrogen, #L12492) for 30 minutes at 37° C. Cells were washed and resuspended in PBS, and analysed by flow cytometry (Attune ™ NxT Flow Cytometer, ThermoFisher Scientific).

### Mitophagy assay

The Cell Meter Mitochondrial Autophagy Imaging Kit Red Fluorescence was obtained from Stratech (#22998-AAT). After sorting, cells were stained with the Mitophagy Red Probe for 30 minutes at 37° C. Cells were washed and resuspended in PBS, and analysed by flow cytometry (Attune ™ NxT Flow Cytometer, ThermoFisher Scientific).

### CD44 analysis

After sorting, cells were incubated with CD44 antibody (APC mouse anti-Human CD44, BD Pharmingen, #559942) for 30 min on ice. Cells were washed and resuspended in 1% BSA PBS, and analysed by flow cytometry (Attune ™ NxT Flow Cytometer, ThermoFisher Scientific).

### ATP assay using Cell-Titer-Glo 2.0

Cell Titer Glo 2.0 was obtained from Promega (#G9242). Ten thousand cells were seeded after sorting in a 96 well plate in complete DMEM medium and incubated with the Cell-Titer-Glo 2.0 Reagent in a humidified atmosphere at 37° C and 5% CO_2_ for 15 min. Luminescence content was evaluated using the Varioskan ™ LUX plate reader (ThermoFisher Scientific).

### Oxidative stress using GSH/GSSG-Glo assay

The GSH/GSSG-Glo Assay Kit was obtained from Promega (#V6611). Ten thousand cells were seeded after sorting into a 96 well plate in complete DMEM medium and incubated with Total and Oxidized Glutathione Lysis Reagent, after incubation cells were treated with the Luciferin Generation Reagent and the Luciferin Detection Reagent following the manufacturer’s recommendations. Luminescence content was evaluated using the Varioskan ™ LUX plate reader (ThermoFisher Scientific). The GSH/GSSG ratio was calculated from the total glutathione and the GSSG measurement.

### Seahorse XFe96 metabolic flux analysis

To evaluate the real-time oxygen consumption rates (OCRs) and the extracellular acidification rates (ECARs) we used the Seahorse Extracellular Flux (XFe96) Analyser (Agilent/Seahorse Bioscience). Fifteen thousand cells per well were plated in the XFe96-well plates after sorting and incubated in a humidified atmosphere at 37° C and 5% CO_2_ for 24 hours. After that, cells were incubated in 175μl/well of XF assay media at 37° C, in non-CO_2_ incubator for 1 hour. During the incubation time, 25μl of 80 mM glucose, 9 μM oligomycin and 1M 2-deoxyglucose (for ECAR measurements) or 10 μM oligomycin, 10 μM FCCP, 10 μM rotenone and 10 μM antimycin A (for OCR measurements) in XF assay media was loaded into the injection ports of the XFe-96 sensor cartridge. Measurements were normalized by protein content (Sulphorhodamine B assay). Data sets were analysed by XFe-96 software.

### Cell cycle analysis

After sorting, cells were incubated with propidium iodide, following the manufacturer’s recommendations (Muse® Cell Cycle Kit, Luminex, #MCH100106) and analysed by FACS (Attune ™ NxT Flow Cytometer, ThermoFisher Scientific).

### Cell migration assay

To measure cell migration, we used Transwell-24 wells with uncoated 8-μm pores transparent PET membranes (Corning, #353097).

Fifteen thousand cells were plated after sorting in the upper chamber of the Transwell in a serum-free DMEM with 1% Penicillin-Streptomycin. The lower chamber contained complete culture medium (DMEM with 10% FBS, 2mM GlutaMAX, and 1% Penicillin-Streptomycin) as chemo-attractant. Cells were incubated in a humidified atmosphere at 37° C and 5% CO_2_ for 16 hours in the migration experiments. Non migrating cells were removed from the upper surface by scrubbing with cotton swabs. Chambers were stained in 0.5% crystal violet for 15 minutes, rinsed in water and examined under a bright-field microscope. Values were obtained by counting four field per membrane (20x objective) and represent the average of at least 3 independent experiments.

### Statistical analysis

All analyses were performed with GraphPad Prism 10. Statistical significance was determined using the Student’s t-test or the ANOVA test; values of less than 0.05 were considered significant. Data are shown as the mean ± SD. All experiments were performed at least three times independently.

### Data availability statement

The original contributions presented in the study are included in the article. Further inquiries can be directed to the corresponding authors.

## Supplementary Material

Supplementary Figures
