# Supplementary material for: Mitophagy and cancer: role of BNIP3/BNIP3L as energetic drivers of stemness features, ATP production, proliferation, and cell migration
Source: Aging (Albany NY). 2024 Jun 4;16(11):9334–49. doi: 10.18632/aging.205939 (PMC11210229; doi:10.18632/aging.205939)
Supplement: Supplementary Figures [file aging-16-205939-s001.pdf]

SUPPLEMENTARY FIGURES

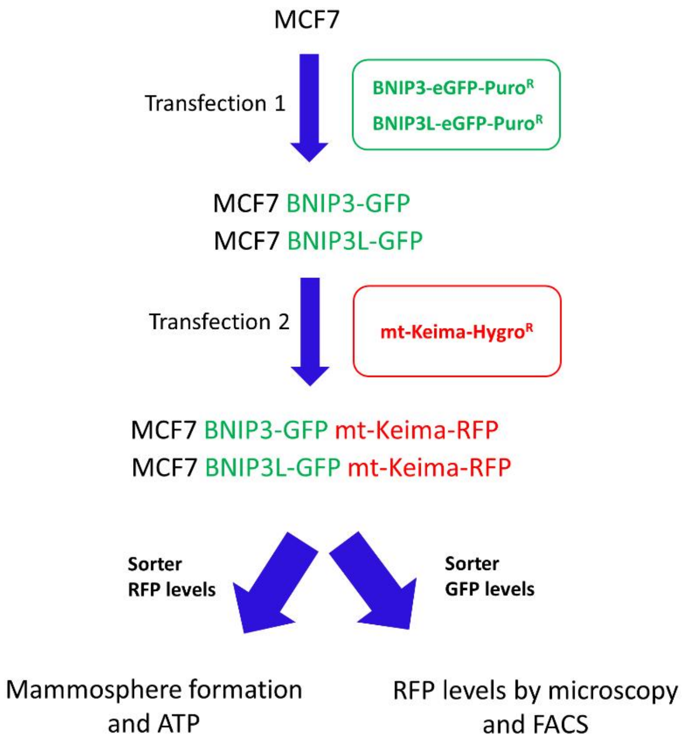

Supplementary Figure 1. Derivation and analysis of MCF7 double-transfectants, harboring both BNIP3(L)-GFP and mt-Keima-RFP.

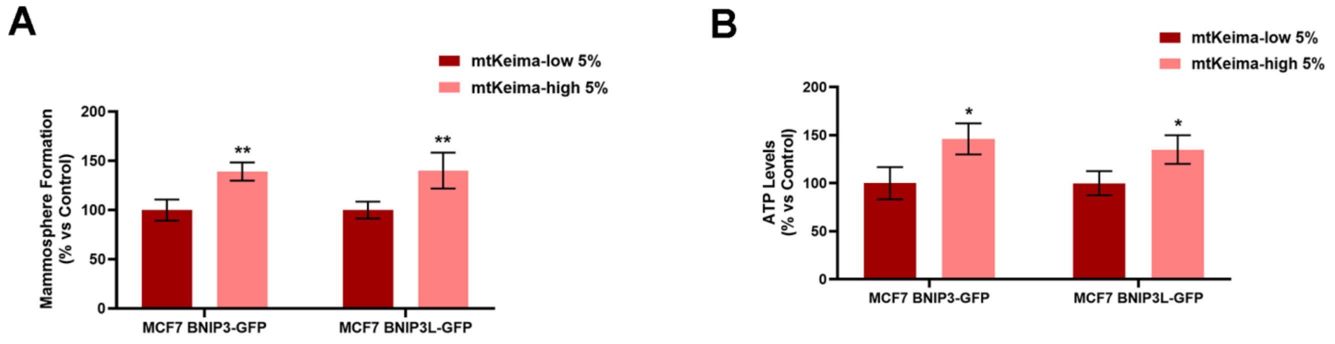

Supplementary Figure 2. mtKeima RFP-high cells show increased ATP levels and elevated mammosphere formation. MCF7 cells stably-transduced with the BNIP3(L)-GFP constructs were subjected to a new transfection with a mt-Keima-Hygro^R construct obtaining two cells lines: MCF7 BNIP3-GFP mtKeima-RFP and MCF7 BNIP31-GFP mtKeima-RFP. Both cell lines were sorting according the RFP levels to isolate the 5% highest RFP (mtKeima-high 5%) and the 5% lowest RFP (mtKeima-low 5%) subpopulations. (**A**) mtKeima-low 5% and mtKeima-high 5% subpopulations were seeded in low-attachment plates for mammosphere assays and analysed after 5 days. Data are shown as mean ± standard deviation (SD) (n = 4). Statistical significance was determined using an unpaired Student's t-test, ** p ≤ 0.01. (**B**) ATP levels were checked by CellTiter-Glo 2.0 Reagent in both subpopulations. Data are shown as mean± standard deviation (SD) (n = 3). Statistical significance was determined using an unpaired Student's t-test, *p ≤ 0.05.
